# Pilot Study of a Hybrid Music Therapy Support Group for Family Caregivers of Older Adults with Dementia

**DOI:** 10.1093/geroni/igaf122.3353

**Published:** 2025-12-31

**Authors:** Jennifer Stull, Sarah Saint Hamilton

**Affiliations:** University of Georgia, Athens, Georgia, United States; University of Georgia, Athens, Georgia, United States

## Abstract

Music can effectively lower perceived levels of stress, anxiety, and depression, which are symptoms commonly experienced by family caregivers. Virtual program delivery may increase access to music-based support for caregivers in underserved areas. The purpose of this study was to examine the feasibility of a hybrid music therapy support group for family caregivers of persons with dementia, as well as evaluate the impact of the support group on perceived stress, anxiety, and depression in family caregivers. Participants (n = 9) attended five hybrid music therapy support group sessions. Participants met in-person in a designated community location while a board-certified music therapist facilitated the session via Zoom. Each session included movement to music, guided breathing experiences, music listening, song discussion, songwriting, and verbal discussion of the session topic. Participants rated their perceived levels of stress, anxiety, and depression on a scale from 0 to 10 at the start and end of each session. Results showed a decrease in mean pre/post-test scores of stress and depression in all five sessions. Ratings of perceived anxiety decreased in all but one session in which the ratings remained the same at the start and end of the session. Additionally, pre-test scores of anxiety decreased 56% from session 1 to session 5, and pre-test scores of stress decreased 52% from session 1 to session 5, showing a decrease in baseline stress and depression levels across the study period. Regarding feasibility, program evaluation revealed minimal issues with hybrid delivery and good audiovisual quality utilizing existing facility infrastructure.

